# Mapping the global origins of soybean: a study using ICP-MS and chemometrics

**DOI:** 10.1038/s41538-025-00630-5

**Published:** 2025-12-09

**Authors:** M. Mar Aparicio-Muriana, Yunhe Hong, Cynthia A. Chilaka, Brian Quinn, Alfredo M. Montes-Niño, Nicholas Birse, Christopher T. Elliott

**Affiliations:** 1 National Measurement Laboratory: Centre of Excellence in Agriculture and Food Integrity, Institute for Global Food Security, School of Biological Sciences, Queen’s University Belfast, Belfast, UK; 2 Microbioticos Paraguay SRL, Arsenales & De las Residentas, San Lorenzo, Paraguay; 3 School of Food Science and Technology, Faculty of Science and Technology, Thammasat University, Khong Luang, Pathum Thani Thailand

**Keywords:** Analytical chemistry, Agriculture

## Abstract

To enhance transparency in the soybean supply chain and help prevent misrepresentation of geographic origin, an analytical method combining ICP-MS with chemometrics was developed. A total of 422 soybean samples were collected from Brazil, the United States, Argentina, China, India, Paraguay and Canada, representing over 95% of global production. The OPLS-DA multivariate analysis model used for classification achieved 98.5% accuracy, with Ni, Na, Mo, Ba, Co, Cr, Cd, Sr, Se, K and Ca identified as key elements for origin differentiation. This approach provides a practical tool for companies and regulators to verify geographic origin, supporting compliance with trade and sustainability requirements and tariff-related controls. Additionally, the ability to differentiate soybean samples from various regions within Brazil and the United States was investigated and preliminary comparisons of meal samples from deforested and non-deforested areas in Brazil revealed elemental differences, suggesting potential environmental influences and highlighting the need for further investigation.

## Introduction

The soybean (*Glycine max*), originating in China and Eastern Asia, was first introduced to Western countries in the 1700s, marking the beginning of its global dissemination^1^. Today, it has become the most economically important legume worldwide, cultivated in numerous countries and serving as a vital source of nutrition. Though primarily used as an animal feed source, soybeans have recently gained prominence in the production of plant-based meat and milk substitutes for human consumption, due to their high protein content^2,3^. In 2019, the Eat-Lancet commission published a report advocating for a shift towards plant-based diets to promote health and sustainability^4^. One of its key recommendations was to increase the consumption of plant-based foods while reducing animal-source foods. Soybeans were highlighted as a crop with a markedly lower environmental impact when assessed on a per-essential amino acid basis, making them crucial for more sustainable diets.

Soybean production takes two primary forms: whole soybean and soybean meal. The former refers to the entire seed, while soybean meal is a byproduct of oil extraction predominantly used as feed for livestock. More than three-quarters of global soybean production is allocated for animal feed, while only 20% is directed to human consumption, including products such as oil, tofu, soymilk, and tempeh, and a small fraction is used for industrial applications (http://news-infographics-maps.net/soy.html).

Global soybean production has experienced remarkable growth, increasing more than tenfold over the past six decades (https://ourworldindata.org/grapher/soybean-production). Since emerging as the leading producer in 2019, Brazil has maintained its dominance, contributing nearly 40% of the world’s soybean production in 2022, followed by the United States (28%), and Argentina (13%). Together with China, India, Paraguay, and Canada, they account for approximately 95% of global soybean production (https://fas.usda.gov/data/production/commodity/2222000). In 2022, global soybean exports surpassed $93 billion, with Brazil and the United States controlling 50% and 36% of the export market, respectively. China stood as the largest importer of soybeans, representing more than 60% of the global import market (https://trendeconomy.com/data/commodity_h2/1201, https://farmdocdaily.illinois.edu/2024/02/the-united-states-brazil-and-china-soybean-triangle-a-20-year-analysis.html).

However, the rapid expansion of the soybean industry, along with other Forest-Risk Commodities (FRC) such as beef, leather, cocoa, coffee, palm oil, wood, and rubber, has been repeatedly linked with deforestation and associated biodiversity loss, greenhouse-gas emissions, and climate change, especially in South America and the Amazon region^5–7^. Agricultural expansion threatens critical ecosystems such as the Amazon rainforest, often described as the “lungs of the earth”^8–11^, and has prompted regulatory responses. For instance, the 2023 European Union Deforestation Regulation (EUDR) requires companies to ensure that imported soybeans are deforestation-free, produced legally, and accompanied by a due-diligence statement^12^. Companies are also expected to implement effective and auditable traceability systems that document the origin of their raw materials. In 2025, Chinese authorities rejected a 300000 t shipment labelled as Argentine after chemical and isotopic analyses revealed a U.S. origin (https://fundacionandresbello.org/en/news/argentina-%F0%9F%87%A6%F0%9F%87%B7-news/china-rejects-300000-tons-of-soybeans-from-argentina-over-suspected-u-s-origin). China continued sourcing soybeans from Brazil to avoid purchasing from the U.S. as a direct results of the trade war between the two nations, demonstrating how geopolitics, tariffs and shifting market conditions can drive mislabelling (https://www.brownfieldagnews.com/news/china-steps-in-for-pork-skips-soybeans-again/). These examples underscore that establishing the country of origin of soybeans is a vital step in detecting and deterring fraud, and that independent, science-based approaches are essential for reliably verifying geographic origin.

The chemical composition of soybeans is significantly influenced by growing conditions, including agricultural practices, such as the use of fertilizers and pesticides, soil properties, and meteorological conditions during crop growth, such as rainfall and temperature^13,14^. Land-use change can further modify soil chemistry; for example, deforestation has been shown to alter nutrient availability, pH, and trace element content in soils, potentially affecting the elemental profile of crops grown in recently cleared areas^15,16^. These variables complicate the ability to determine the geographical origin of soybeans using a single measurement. A recent review assessed the existing analytical methods for tracing soybean origin, identifying gaps in current techniques^17^. It recognized the potential of DNA techniques, Raman spectroscopy, and hyperspectral spectroscopy for deducing soybean provenance. Spectroscopy-based approaches, though widely used, still present certain challenges such as small and non-representative sample size for modelling^18^, and the absence of quality control (QC) samples or certified reference materials (CRM), which can result in instrumental errors that outweigh the true inter-group differences^19^. In addition, variables such as sample moisture content and particle size can substantially influence spectroscopic results, yet many established analytical methods overlook these considerations^20^.

Chromatography techniques, especially when coupled with mass spectrometry, have demonstrated great potential in determining the origin of food products^21,22^. According to the existing literature, gas chromatography (GC) and liquid chromatography (LC) are particularly effective for quantitative analysis of functional components in soybeans^21,23^. Additionally, stable isotope analysis has proven potential in determining the geographic origin of soybeans among several widely traded commodities^24–28^. For instance, Wu et al. explored the potential of element analyser-stable isotope ratio mass spectrometry (EA-SIRMS) to analyse the carbon and nitrogen isotopic composition of soybean samples from three Chinese regions^29^, and Zhou et al. combined stable isotope analysis, non-metallic element content, and mineral element content with chemometric techniques to classify soybean samples from China, the United States, Argentina and Brazil^30^. While many of the analytical methods reported in the literature show promise, they often suffer from limitations such as restricted sample size or geographic scope^31,32^, making their widespread application for routine testing questionable. This highlights the need for more comprehensive and robust techniques to reliably trace soybean origin.

Inductively coupled plasma mass spectrometry (ICP-MS), first introduced by Houk et al. and commercially available since the 1980s^33,34^, has become a valuable instrumental method for trace element analysis due to its unique attributes. These include the capability to analyse multiple elements simultaneously, low detection limits, a broad linear dynamic range, and efficient sample throughput, making it particularly valuable in the food industry^35^. Its capacity to detect minute differences in elemental profiles – often resulting from environmental and geological variations – enables precise differentiation of geographic origins^36–38^. ICP-MS allows for the simultaneous analysis of several elements in a single run, generating a detailed elemental profile and quantifying both major and trace elements at very low (sub-ppm) concentrations^39^. The technique has a low cost per sample, is rugged, and relatively rapid. Additionally, its technical features make it easy to accredit to an international standard and distribute to other laboratories across a wide geographical area^40^. For effective interpretation of complex analytical datasets, advanced statistical tools are often required. Multivariate analysis (MVA), also known as chemometrics when applied to chemical data, provides these tools by enabling data pre-processing, variable selection, pattern recognition, data dimension reduction, quantitative calibration, and multi-platform data fusion^41^. Applying MVA to the complex elemental fingerprints generated by ICP-MS fully exploits the information contained in the data and improves the robustness and accuracy of origin discrimination in food and feed commodities^30,39,42,43^.

Previous studies have demonstrated the effectiveness of ICP-MS combined with MVA for classifying soybeans based on geographic origin. For example, Cui et al.^14,44^ analysed soybean samples from specific provinces in China (12-17 elements; 91-116 samples; 90-93% accuracy)^14,44^; Nguyen-Quang et al.^45^ worked with small sets from Vietnam, Canada, the United States and Brazil (33 elements; total 38 samples)^45^; and Hidalgo et al.^46^ examined three Argentine provinces (20 elements; 120 samples; 99% accuracy)^46^. However, these studies focused on regions within a single country or a small number of countries and relied on a relatively small sample size.

The present study aims to develop the most comprehensive ICP-MS combined with MVA approach to date for determining soybeans’ geographical origin. For the first time, soybeans from all major growing regions worldwide were analysed. A total of 422 samples were collected from multiple farms across seven top-producing countries over four harvest years, capturing both spatial and temporal variability by quantifying forty elements. This thorough and detailed dataset enhances traceability and supports compliance with regulatory requirements for the verification of geographic provenance.

## Results

### ICP-MS method validation

The accuracy of the ICP-MS method was evaluated through both CRM recovery checks and spike recovery experiments. CRM recovery checks were conducted throughout the study using an organic soybean meal reference material, closely matching the matrix of the samples. These checks were conducted at the start and end of each sample batch for elements with manufacturer-reported concentrations, including Cu, Al, K, Fe, Ca, Mg, Na, B, Zn, and Mn. The recovery rates for the monitored elements were within 100 ± 30%, except for Al, which was within 100 ± 40%.

Complementing this, a spike recovery study was conducted using a soybean sample from Canada to assess potential matrix effects. The unspiked sample was first analysed to determine baseline element concentrations, after which triplicate spiked samples were prepared at varying concentrations depending on the element’s original concentration. The recoveries were calculated by comparing the measured concentration in the spiked samples to the expected values. The observed recoveries were within 100 ± 20%, with most elements falling within 100 ± 10%, confirming minimal matrix interference and high analytical accuracy. Full recovery data are presented in Table S1.

The robustness of the elemental measurements was further evaluated by comparing the concentrations of all monitored elements detected in the CRM at the start and at the end of each sample batch. This evaluation included CRM analyses across multiple batches and days. After analysing over 100-120 samples per batch, the percentage difference between the concentrations measured was found to be less than 14.2% for all elements included in the model, with an average percentage difference of 4.50%, indicating strong analytical consistency. Figure S1 and Table S2 present the concentration comparisons and percentage differences.

Instrument precision was assessed by evaluating both intra-day (repeatability) and inter-day (reproducibility) variability using two separate standard mixes: one containing Na, Mg, Ca, Fe, and K at a concentration of 195 µg/L, and another containing the remaining elements at 3.12 µg/L. For intra-day precision, three consecutive replicate measurements of each standard mix were taken on the same day, while inter-day precision was evaluated by taking three replicate measurements across three different days. Precision was expressed as the relative standard deviation (RSD) for each element. The results showed that the average RSD for Na, Mg, Ca, Fe, and K was less than 7.10% for both intra-day and inter-day precision, while for the rest of the elements, the average RSD was less than 5.30%. None of the elements exhibited an RSD exceeding 17.0%, indicating strong precision.

To minimize systematic errors or analytical bias, samples from different countries were included in each batch, and their order was varied across batches. Sample blanks were also included in every batch to monitor for contamination or baseline shifts.

Detection limits were established for all 40 elements. Calibration curves showed excellent linearity, with coefficients of determination (R) greater than 0.9995. Instrument detection limits (IDLs) were calculated as three times the standard deviation of ten replicate measurements of the calibration blanks. Method detection limits (MDLs) were obtained by applying a 200-fold dilution factor to the IDLs. Full IDL and MDL values can be found in Table S3.

### Exploratory multivariate analysis of soybean elemental profiles

A total of 422 soybean samples from the seven major producing countries—Brazil, the United States, Argentina, China, India, Paraguay and Canada—were analysed in this study. To validate this methodology, an organic soybean meal CRM was included with each batch to ensure the method’s reliability and stability. Each sample was prepared in triplicate and analysed using ICP-MS, resulting in a substantial dataset of elemental information.

**Fig. 1:**
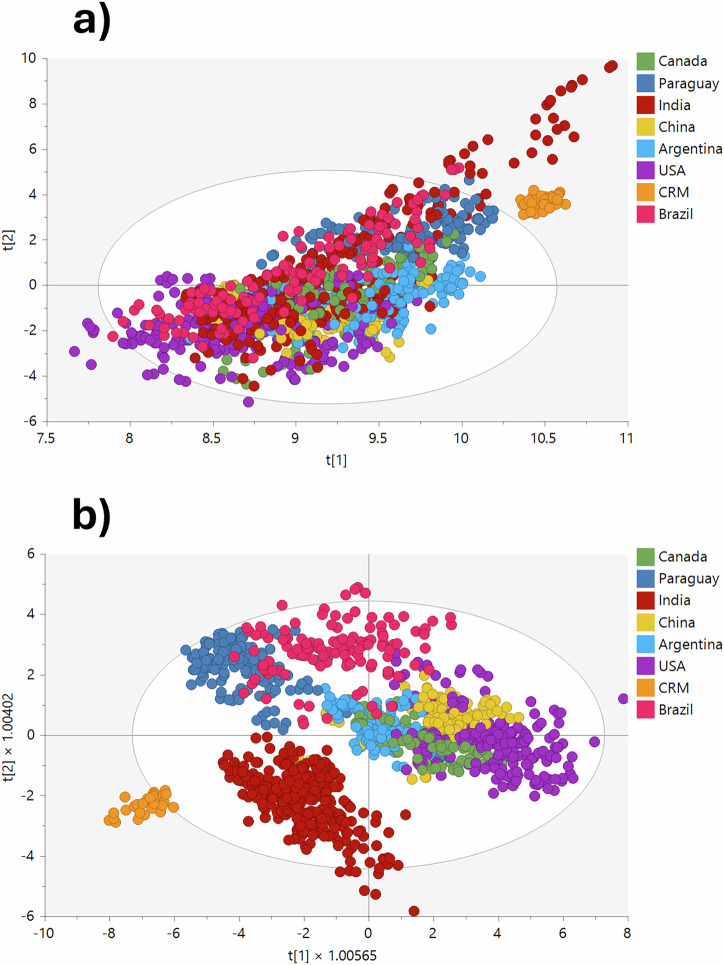
Classification of soybeans origin across seven countries and certified reference material (CRM) with three replicates per sample. **a** PCA score scatter plot showing classification among seven countries and CRM. **b** OPLS-DA score scatter plot showing classification among seven countries and CRM.

An initial exploratory analysis was conducted using principal component analysis (PCA) on all three replicates for each sample to examine the data across the eight groups, assess the global structure of the dataset, identify patterns and check for potential outliers. The unsupervised PCA model derived from the ICP-MS data provided an overview of the entire dataset, showing substantial differences between the eight groups (Fig. 1a). The accuracy and reliability of the model were evaluated through the R^2^X and Q^2^ parameters. R^2^X assessed the proportion of the variance in the elemental concentrations (X) explained by the model, while Q^2^ evaluated the model’s predictive quality and the fraction of the variation in the soybean sample classifications (Y) that could be reliably predicted. The model demonstrated strong performance, with an R^2^X = 0.976 (A = 6, where A is the number of principal components) and good explanatory and predictive abilities, with a Q^2^ value of 0.947.

The inclusion of CRM alongside the other groups further confirmed the reliability and stability of the instrument, analysis method, and data processing technique. The PCA-class model for the CRM, with R^2^X = 0.996 and a Q^2^ = 0.995, reflected a high level of consistency and credibility in the data (Figure S2). Orthogonal partial least squares discriminant analysis (OPLS-DA) models (using UV scaling) were built using the whole dataset comprising elemental information of seven soybean origins, with the CRM group also being included to monitor model performance (Fig. 1b). The OPLS-DA model demonstrated acceptable performance with R^2^X, R^2^Y, and Q^2^ values of 0.803, 0.685 and 0.679, respectively. The high R^2^X indicated a good fit for the model, while R²Y and Q² suggested strong predictive ability. As OPLS-DA is a linear model^47^, it tends to introduce bias towards the group or groups with a larger representation when there is a substantial discrepancy in the number of instances across subsets. While initial models were built using all available samples, for this study the intention was to minimize sample size variation across the different groups to prevent overfitting to larger groups and mitigate the risk of diminished model performance. In the subsequent analyses described in this manuscript, a balanced representation was achieved by selecting approximately 50 samples per country, further reducing this risk and preserving model accuracy.

### Discrimination of soybean geographical origins using elemental profiles

After confirming the initial group separation through PCA and validating the method’s stability with the CRM, a more comprehensive analysis was conducted to refine the identification of soybean geographical origin based on elemental fingerprinting. For this purpose, the CRM group was excluded, and the analysis focused on the seven major soybean-producing countries. To ensure balanced representation across countries, a subset of 330 soybean samples was selected from the full dataset. The selection was guided by the goal of achieving a roughly equal number of samples per country, resulting in 42 samples from Brazil, 50 from the United States, 50 from Argentina, 50 from China, 50 from India, 50 from Paraguay, and 38 from Canada. The elemental profiles obtained from the ICP-MS analysis were averaged across the three replicates for each sample. The resulting dataset provided a comprehensive representation of the elemental profiles, which was subsequently used to evaluate the geographical differentiation of soybean samples through both unsupervised and supervised multivariate analyses.

**Fig. 2:**
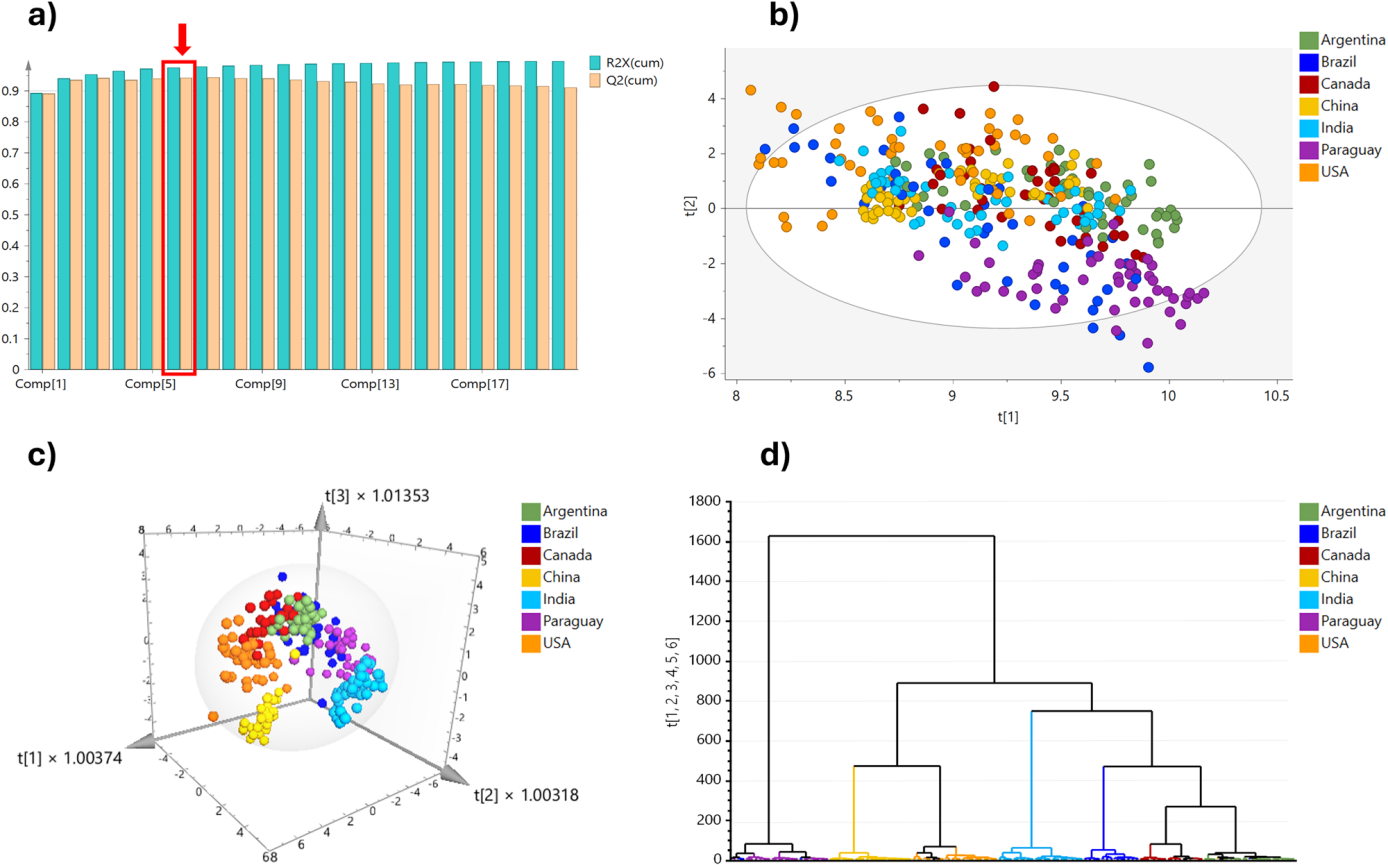
Classification of soybean origins across seven major producing countries. **a** Cumulative R²X and Q²X plot per component for the PCA model. **b** PCA score scatter plot for the seven countries. **c** 3D OPLS-DA score scatter plot for the seven countries. **d** Hierarchical clustering analysis (HCA) based on the OPLS-DA model.

Unsupervised PCA model using six principal components yielded the best equilibrium between explained variance and predictive capacity across the seven groups (Fig. 2a). PCA was performed on the entire dataset to explore natural groupings and underlying variance structure prior to building supervised classification models. The PCA score plot revealed distinct separations among the groups across PC1 and PC2 (Fig. 2b). The R^2^X value of 0.978 indicated that the PCA model was well-fitted, and Q^2^ value of 0.948 reflected strong explanatory and predictive performance, reaffirming the reliability of the dataset for further supervised analysis.

Subsequently, a supervised OPLS-DA model was built to assess the separation patterns previously observed in the PCA. To ensure robustness and avoid overfitting, the subset of 330 samples was divided into two sets using a stratified random sampling approach: 80% of the samples were used to train the model, while the remaining 20% were held out as a test set to evaluate the model’s performance. This training/test approach enabled a thorough assessment of the model’s generalizability in classifying soybean origins based on their elemental fingerprints. The OPLS-DA model was developed using the training set, and its accuracy and robustness were assessed by evaluating its ability to predict the origin of samples in the test set based on the elemental data. The separation among the seven countries was visible the in 3D OPLS-DA score plot (Fig. 2c). The values R^2^X = 0.784, R^2^Y = 0.727, and Q^2^ = 0.679 indicated good fit and predictive power. To further explore relationships among the samples, a hierarchical clustering analysis (HCA) was performed. As depicted in Fig. 2d, the HCA revealed distinct clusters that correspond to each country’s elemental profiles, indicating significant similarities in the composition of samples from the same origin. This clustering supports the findings from the OPLS-DA model, which demonstrate the effectiveness of elemental profiling in distinguishing between soybean origins.

In addition, a 100-time permutation was performed to assess the validity of the OPLS-DA model. The results demonstrated that the intercept values of R^2^Y and Q^2^ from the permuted models on the left were lower than those obtained from the original data on the rightmost side (Figure S3). Notably, the intercept value of Q^2^ in the permutation plot was below zero, further confirming that the developed OPLS-DA model was not overfitted.

The performance of the model was then evaluated using the test set. The results are summarized in the confusion matrix (Table 1), which illustrates the model’s performance in classifying the samples by comparing the predicted origins by the OPLS-DA model against the true ones. Each cell in the matrix represents the number of samples that were classified into each category, allowing for an assessment of both correct and incorrect predictions. The model’s predictive performance was measured by its accuracy rate, defined as the proportion of correctly classified samples out of the total test set. Achieving a 98.5% accuracy rate underscores the model’s high efficacy in correctly identifying sample origins.

**Table 1:** Confusion matrix for classifying soybean origins using the OPLS-DA model

	Members	Correct	Argentina	Brazil	Canada	China	India	Paraguay	USA
Argentina	10	100%	10	0	0	0	0	0	0
Brazil	8	87.5%	0	7	0	0	1	0	0
Canada	8	100%	0	0	8	0	0	0	0
China	10	100%	0	0	0	10	0	0	0
India	10	100%	0	0	0	0	10	0	0
Paraguay	10	100%	0	0	0	0	0	10	0
USA	10	100%	0	0	0	0	0	0	10
Total	66	98.5%	10	7	8	10	11	10	10

### Multivariate exploration of elemental profiles for soybean origin discrimination

To explore elemental patterns that contribute to the differentiation of soybean samples from the seven major producing countries, heatmap visualization, variable importance in projection (VIP), pairwise comparison, and loading plots were analysed and discussed. These tools are valuable for highlighting trends and the influence of elements on geographical distinction among the samples.

**Fig. 3:**
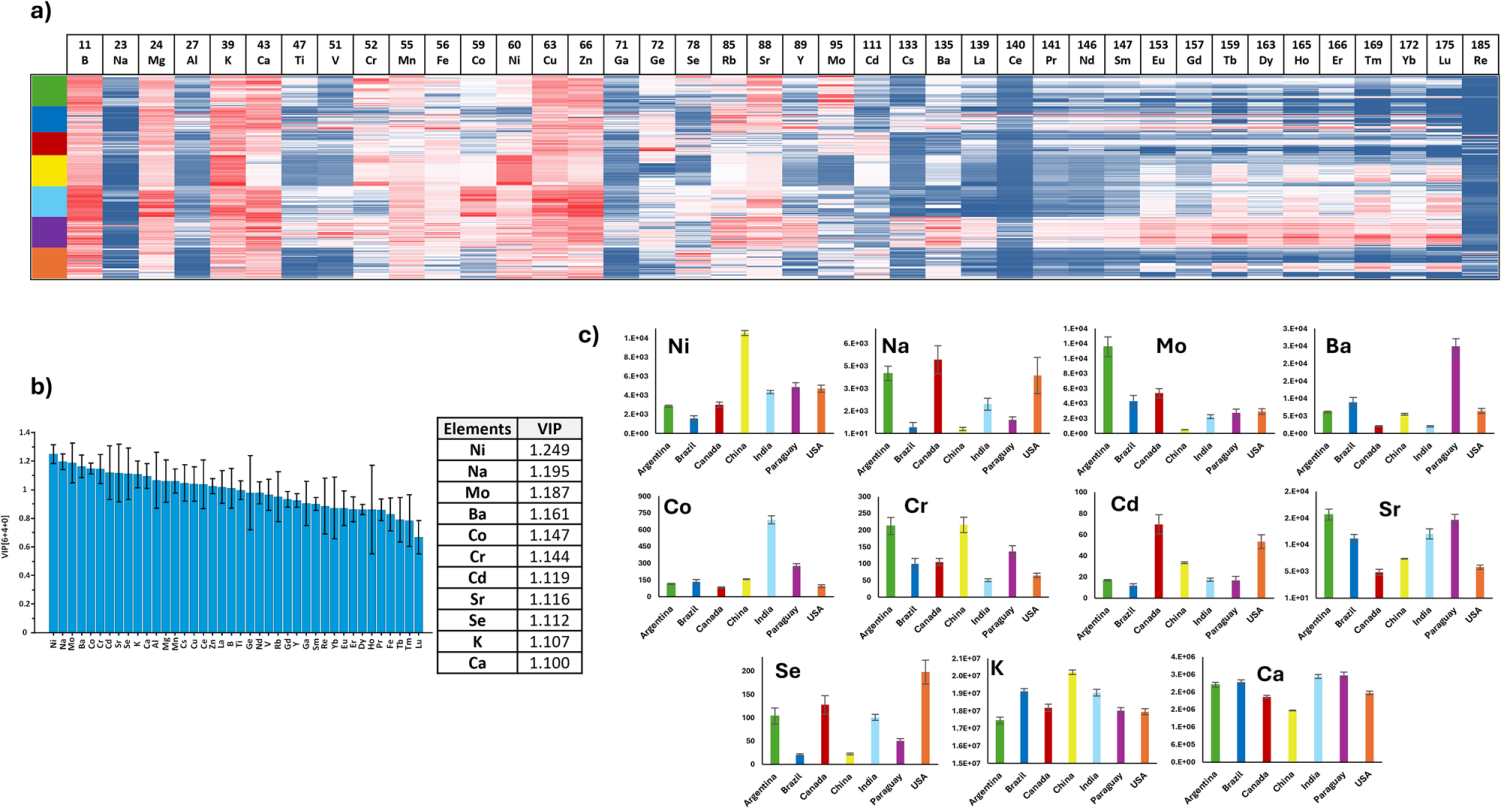
Elementomics analysis for distinguishing soybean origins among the seven major producing countries. **a** Heatmap of Min-Max-normalized concentrations of 40 elements in soybeans from Argentina (green), Brazil (dark blue), Canada (red), China (yellow), India (light blue), Paraguay (purple) and the USA (orange). **b** VIP analysis indicating the contribution of various elements in differentiating soybean origins. Some influential elements identified include Ni, Na, Mo, Ba, Co, Cr, Cd, Sr, Se, K, and Ca. **c** Pairwise comparisons of selected marker element concentrations across countries. Error bars represent the standard error.

As an initial indicator of distinct elemental patterns associated with different soybean origins, a heatmap was constructed using Min-Max normalized elemental concentrations across all samples. This method effectively illustrates trends in the data, as it shows the relative abundance of elements across the samples. In Fig. 3a, the colour gradient from red to blue reflects the transition from high to low elemental concentration, with visible differences that may point to geographical specificity in the elemental profiles. These visual contrasts serve as a first indication of distinct elemental patterns associated with different soybean origins.

**Fig. 4:**
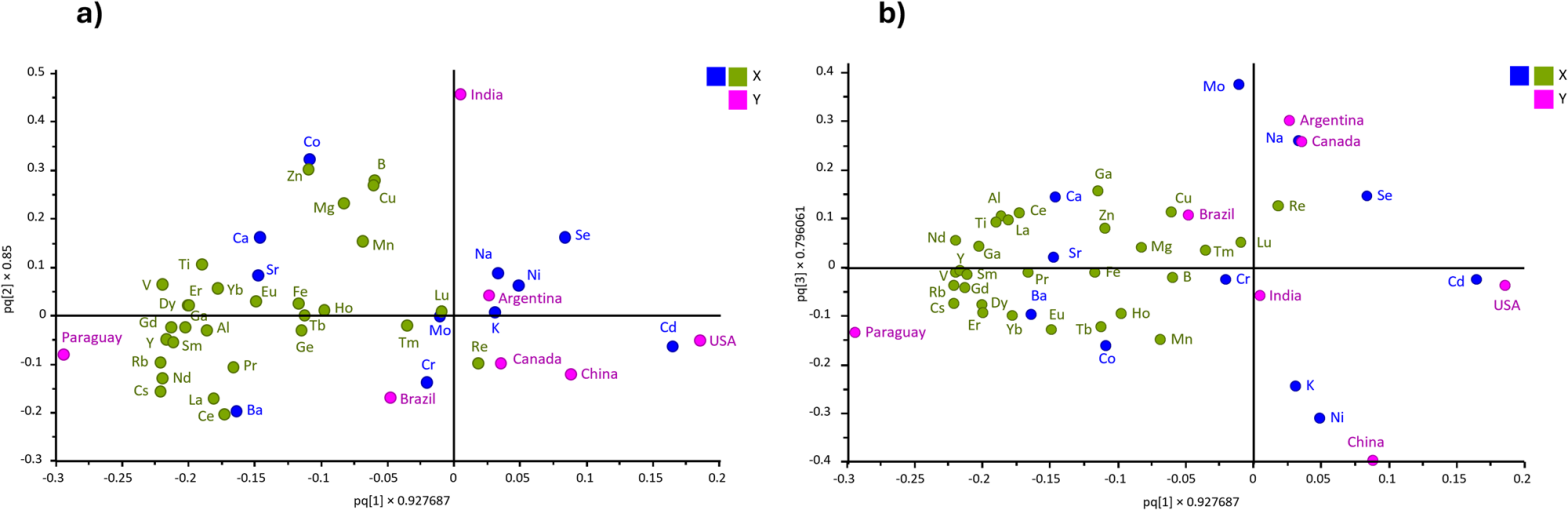
Loading plots from the OPLS-DA model illustrating the contribution of elemental variables to the geographic separation of soybean samples. **a** Loading plot for component 1 vs. component 2. **b** Loading plot for component 1 vs. component 3. Each dot represents a variable (element), with dark blue indicating the selected marker elements based on VIP scores, green representing the remaining elements, and pink denoting the sample group centroids.

VIP analysis, in comparison, quantifies the contribution of each element to the differentiation between the groups, with higher VIP scores indicating a greater contribution of elements to the overall separation (Fig. 3b). A threshold value of 1.1 was used to select influential elements, as it provided a balanced number of markers (11 elements) for interpretation; using a lower threshold (e.g., 1.0) would include too many elements (around half of them), while a higher threshold (e.g., 1.2) would be too restrictive, leaving only one element. Among the more influential elements exhibiting distinctive patterns useful for differentiating soybean origins were Ni, Na, Mo, Ba, Co, Cr, Cd, Sr, Se, K, and Ca. However, elements below this cutoff also contribute to the overall classification performance and should not be disregarded. To further evaluate and visualize the relevance of these top-ranked markers, a pairwise comparison analysis was conducted. This targeted analysis highlighted the most prominent regional contrasts in elemental composition across the seven countries, supporting their potential discriminatory power (Fig. 3c). Additionally, loading plots visualize the contribution and directionality of each element in the model’s component space, providing further insight into these patterns (Fig. 4a, b). China was particularly distinguished by elevated levels of Ni and Cr, and notably low concentrations of Mo. Figure 4b shows how Ni is oriented in the direction of the Chinese cluster, indicating a strong positive association, while Mo is positioned in the opposite direction, reflecting its negative correlation with Chinese samples. Paraguay stood out due to high Ba and Sr levels and relatively low concentrations of Se, Na, and Cd. This contrasts with the profiles of Canada and the USA, which showed elevated levels of Se, Na, and Cd, along with lower Ba and Sr. These opposing trends are supported by the loading plots (Fig. 4a, b), where the vectors for Ba and Sr point toward Paraguay, while those for Se, Na, and Cd are directed toward Canada and the USA, located far from the Paraguayan cluster, reinforcing the observed geographical divergence. Argentina was characterized by high levels of Mo and Sr, and among the lowest Co concentrations. In contrast, India presented the highest Co concentrations and the lowest Ba content relative to other countries. These opposing associations are also visible in Fig. 4a, where Co appears close to the Indian cluster, indicating a positive association, whereas Ba is positioned on the opposite side, consistent with its reduced presence in Indian samples. Brazil showed relatively low levels of Ni, Cd, and Se but did not show extreme high values, placing it in a more intermediate position across most elemental trends. This balanced profile is reflected in the loading plots, where Brazilian samples occupy a relatively central region, not strongly associated with the directional vectors of the most discriminatory elements. Altogether, the integration of VIP scores, pairwise comparisons, and loading plot interpretations supports the robust role of these elements in explaining the geographic variation in soybean elemental profiles. These trends also align with the spatial groupings observed in the 3D OPLS-DA model (Fig. 2c).

The observed patterns in elemental concentrations across different geographical regions are likely the result of several interconnected factors. Regional soil composition is a primary factor, as it influences the bioavailability of elements to plants. Additionally, local climatic conditions, such as temperature, rainfall, and sunlight, can impact plant growth and metabolism, thus affecting elemental uptake., Variations in agricultural practices, including fertilization, irrigation, and soil management, could also contribute to the distinct elemental profiles observed between countries. Finally, genetic differences in soybean varieties grown in different regions may lead to differences in elemental accumulation, further distinguishing samples by their geographical origin. The results of this study demonstrate the reliability of elemental fingerprinting combined with MVA, particularly OPLS-DA, in accurately determining the geographical origin of soybeans.

### Regional discrimination of soybeans in Brazil and the USA

Following the effective distinction of soybean samples sourced from the leading producing countries, further modelling was undertaken to explore whether elemental profiling could distinguish soybeans from more localized regions within individual countries, specifically within the United States and Brazil. For this exploration, 67 soybean samples from eleven states in the United States and 31 samples collected from six states in Brazil were analysed. Due to the limited number of samples per state, robust state-level discrimination was not feasible. To address the limitation of sample size, the soybean samples were grouped into broader regions: Central USA and East USA for the American samples, and Cerrado and Atlantic Forest for Brazil. This grouping reflects not only political boundaries but also distinct environmental and agricultural characteristics. In Brazil, the Cerrado biome, including states such as Minas Gerais, Goiás, and the Federal District, has a humid-tropical climate with a well-defined rainy season. This region is significantly linked to deforestation and environmental degradation (https://gfr.wri.org/forest-extent-indicators/deforestation-agriculture). On the other hand, in the Atlantic Forest region, which includes states such as São Paulo, Paraná and Rio Grande do Sul, where the weather is predominantly subtropical (cold and dry), soybean productivity tends to be below the average for Brazil (https://www.czapp.com/explainers/soybean-in-brazil/, https://www.czapp.com/analyst-insights/5-tips-to-understand-brazils-soybean-production/). This region’s remaining forested areas are under much stricter protection laws than the Cerrado biome, limiting new agricultural expansion.

In the United States, Central states benefit from a combination of favourable climate conditions, fertile soils, and well-established agricultural infrastructure that supports the large-scale cultivation of soybeans, while Eastern regions feature more mixed agricultural systems (https://worldpopulationreview.com/state-rankings/soybean-production-by-state). Grouping these samples based on broader regions allowed us to explore elemental differences more effectively. Detailed information on the state origins, number of samples and grouping strategy can be found in Tables S4 and S5.

**Fig. 5:**
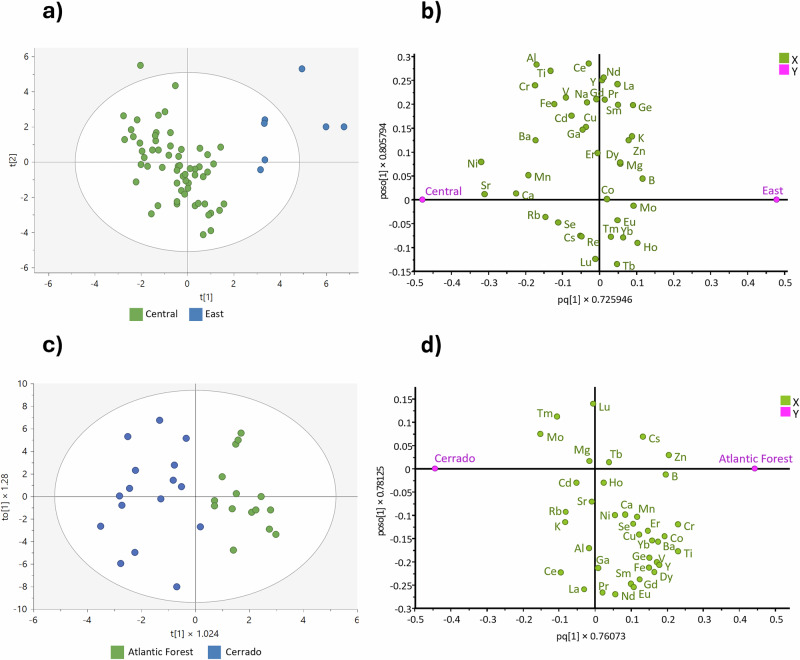
Regional classification of soybean origins using OPLS-DA models. **a** OPLS-DA score scatter plot for regional analysis in the USA. **b** OPLS-DA loading plot for regional analysis in the USA. **c** OPLS-DA score scatter plot for regional analysis in Brazil. **d** OPLS-DA loading plot for regional analysis in Brazil.

The PCA models did not reveal meaningful separation between Central and East regions in the USA, or the Cerrado and Atlantic Forest regions in Brazil. However, the OPLS-DA models showed significant clustering between the broader regional groups in the USA (Fig. 5a) and Brazil (Fig. 5c). To complement these findings, the corresponding loading plots are presented alongside the score plots (Fig. 5b, d), offering an overview of the variables driving the observed separation. In the USA model, Ni and Sr were identified among the most influential elements based on the VIP analysis, positioned close to the Central USA cluster in the loading space. In Brazil, Ti and Cr emerged as key contributors. While these findings are preliminary due to the limited sample size, they provide valuable insights and support the potential of elemental profiling for sub-national geographic classification of soybean samples.

### Preliminary study on differentiating soybean meal samples from deforested and non-deforested areas

Building upon the insights gained from the elemental profiles of soybeans across various geographical origins, a preliminary examination was conducted to explore whether elemental analysis can differentiate soybean meal samples sourced from deforested and non-deforested areas within Brazil. Given the increasing concern regarding the environmental impacts of agriculture on deforestation, understanding potential elemental markers linked to deforestation-related soil changes is of significant interest. Soil disturbances due to deforestation can alter pH, nutrient availability, and microbial activity, potentially affecting elemental uptake in crops. However, multiple factors beyond deforestation, such as soil type, fertilization, and climate, can also contribute to elemental composition variations^48^.

To explore this hypothesis, a total of 24 soybean meal samples were analysed: six from deforested regions and eighteen from non-deforested regions. Due to the limited and imbalanced sample size, an exploratory data analysis approach was adopted to assess whether detectable differences exist; PCA and OPLS-DA were employed for pattern recognition.

**Fig. 6:**
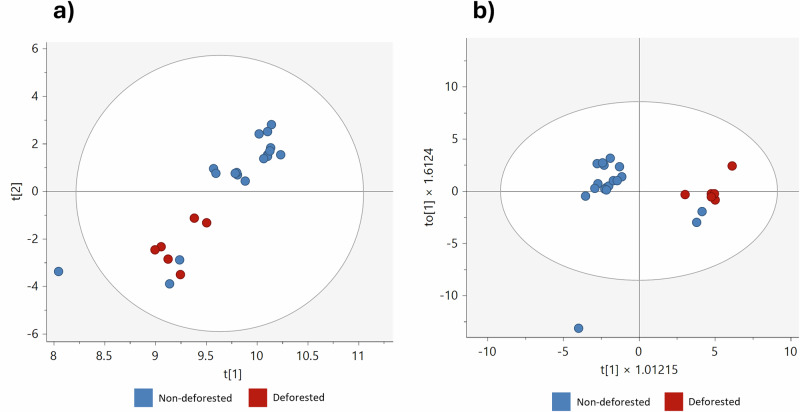
Multivariate analysis of elemental concentrations in soybean meal samples from deforested and non-deforested areas in Brazil. **a** PCA score scatter plot. **b** OPLS-DA score scatter plot.

The PCA plot suggested some degree of separation between samples from deforested and non-deforested regions (Fig. 6a). The PCA model showed high R²X (0.985) and Q² (0.957), indicating that most variance in the dataset was captured; however, as PCA is an unsupervised technique, these results should be interpreted cautiously. The OPLS-DA model yielded R²X, R²Y, and Q² values of 0.558, 0.687, and 0.246, respectively, suggesting limited predictive accuracy and robustness, likely due to the small dataset and imbalanced group sizes (Fig. 6b). These findings highlight that while elemental composition differences may exist, further validation is required before drawing definitive conclusions.

Interestingly, two samples labelled as non-deforested clustered within the deforested group, suggesting potential misclassification. This misclassification could stem from an insufficient sample representation within the model or indicate that these samples originated from deforested areas but were mislabelled. While this preliminary study suggests that elementomics coupled with MVA may offer a viable approach for differentiating soybean meal samples based on deforestation status, further refinement is needed to confirm its reliability.

To strengthen future studies, an expanded and more balanced sample set is necessary to enhance statistical reliability. Additionally, incorporating metadata such as soil composition, agricultural practices, and climate conditions could help account for potential confounding factors. While this study presents preliminary evidence of elemental composition differences related to deforestation status, it should be viewed as an initial exploratory step rather than a conclusive determination. Further research with a larger dataset and external validation is needed to establish a robust and generalizable model for differentiating soybean meal samples based on deforestation impact.

## Discussion

In this study, a comprehensive analysis was conducted on soybean samples from the seven major producing countries using ICP-MS and MVA techniques to classify and differentiate the samples based on their geographical origin. This is the first study that encompasses such a broad scope of countries and growing seasons while analysing a large sample set, with 422 samples examined and 330 samples used to build a balanced country-level classification model. A total of 40 elements were quantitatively monitored to capture the elemental profiles, and a detailed validation process including CRM recovery checks, spike recovery studies, precision assessments, and batch consistency evaluations to ensure the reliability and robustness of the results.

The multivariate analysis of elemental profiles demonstrated a robust capacity to discriminate soybean samples based on their geographical origin. The PCA model exhibited strong explanatory and predictive performance, as indicated by high R²X (0.978) and Q² (0.948) values. Further refinement using the OPLS-DA model resulted in a 98.5% classification accuracy on test data, confirming the model’s reliability in predicting sample provenance.

Among the 40 analysed elements, a subset—including Ni, Na, Mo, Ba, Co, Cr, Cd, Sr, Se, K, and Ca—emerged as key discriminators of origin, as evidenced by high VIP scores and consistent loading plot orientations. These elements showed distinct geographic trends, with specific countries such as China, Paraguay, and India exhibiting characteristic profiles. Altogether, the integration of heatmaps, VIP scores, pairwise comparisons, and loading plots provides a comprehensive framework for understanding elemental variability and its relationship to geographical origin.

Beyond country-level classification, variations within soybean samples from the same country were explored, particularly for the United States and Brazil. Initial findings demonstrated some regional separation, though further samples are needed to improve the statistical confidence and capture broader intra-country variability. A preliminary study on soybean meal samples from deforested and non-deforested areas suggested differences in elemental composition between the two groups, as indicated by the PCA and OPLS-DA models. While these results highlight the potential of elemental analysis for environmental monitoring and sustainable soybean origin verification, a larger and more balanced sample set is necessary to enhance the reliability and generalizability of these findings.

Overall, this study demonstrates the effectiveness of ICP-MS-based elementomics combined with chemometric analysis as a powerful tool for authenticating soybean origin. The approach offers promising commercial applications for businesses seeking to enhance supply chain transparency and verify deforestation-free sourcing, ultimately contributing to rainforest conservation efforts. Considering the results obtained in this study and the promising differentiation observed within Brazilian samples, a future phase of this research will focus on a more detailed regional exploration within Brazil—given the country’s pivotal role in the global soybean market and its environmental implications—by including a broader and more representative sample set of the major producing regions in Brazil to enhance regional classification.

## Methods

### Chemicals

Nitric acid (67-69%), hydrogen peroxide (30%) and hydrochloric acid (37%) were purchased from VWR (Lutterworth, UK). Indium (8500-6946) and bismuth (8500-6936) internal standards, instrumental tuning solution (5190-0465), and multielement calibration standard solutions including environmental calibration standard (5183-4688), calibration standard-1 (8500-6944), calibration standard-2A (8500-6940) and calibration standard-4 (8500-6942) were supplied by Agilent Technologies (Santa Clara, CA, USA). Soybean meal organic analytical standard was obtained from Elemental Microanalysis (Okehampton, UK) and 18.2MΩ deionised water was produced by a Millipore Integra 3 system (Merck-Millipore, Billerica, MA, USA).

### Sample collection and preparation

A total of 422 yellow soybean (*Glycine max*) samples from the seven major producers contributing to 95% of global production—Brazil (*n* = 42), the United States (*n* = 79), Argentina (*n* = 50), China (*n* = 59), India (*n* = 104), Paraguay (*n* = 50) and Canada (*n* = 38)—were obtained and analysed. Samples were provided by trusted suppliers either as whole soybeans or in powdered form. In Argentina, samples originated from different cities in three key soybean-producing provinces: Tucumán, Santiago del Estero, and Salta. Brazilian samples were sourced from several key producing states, including Minas Gerais, São Paulo, Paraná, Goiás, Rio Grande do Sul, and Federal District. For China, approximately half of the samples were sourced from Heilongjiang Province, one of the country’s most important soybean-growing areas in the northeast, with the remaining samples being sourced from other key soybean-growing provinces such as Liaoning, Shaanxi, Jiangsu, Sichuan, Shandong, Anhui, and Hunan. Indian samples were sourced from districts in the states of Maharashtra (Buldhana, Beed, Latur, Hingoli, Akola, and Washim) and Madhya Pradesh (Indore and Ujjain). Paraguayan samples were sourced from locations including Antequera, Concepción, Ñacunday, Puerto Rosario, and Asunción; however, most were associated with individual silos or sourcing entities (e.g., Silo Mariani, Silo R. Balbuena, and Silo AgroferTil) reflecting different commercial batches. USA samples were sourced from several states such as South Dakota, Missouri, Illinois, Arkansas, Nebraska, Iowa, Ohio, Kansas, Maryland, and North Carolina. For Canada, no information was available on their geographic origin within the country.

In addition, six Brazilian soybean meal samples provided by a commercial importer—labelled as originating from deforested areas—and 18 samples labelled as from non-deforested areas were analysed; however, no further specific location details were available for these samples. The samples were stored at −20 °C before analysis and fully thawed at room temperature prior to sample preparation and analysis. The soybean samples were ground and homogenized using a compact kitchen food processor (Cuisinart, Stamford, CT, USA) before sample treatment. Three replicates were analysed from each sample.

The following approach was used to digest soybean samples: 100 ± 5 mg of ground soybean sample was weighed and transferred to a 50-mL polypropylene tube before adding 2 mL of nitric acid. The sample was left in a fume hood for digestion for at least 15 h. Then, 2 mL of hydrogen peroxide was added to each sample before microwave digestion using a Mars 6 system (CEM, Matthews, NC, USA). The digestion protocol was as follows: the samples were heated from room temperature to 54 °C in 5 minutes, held at 54 °C for 15 minutes, then heated to 65 °C over the next 5 minutes, and held at 65 °C for 10 minutes. Finally, the samples were heated to 95 °C and kept at that temperature for 30 minutes. After cooling, the tubes were filled to 20 g with deionised water (18.2MΩ) with 0.5% HCl using a VWR SE622 balance (VWR, Leuven, Germany). Blanks were included to monitor contamination.

### ICP-MS analysis

Samples were analysed for 40 elements using an Agilent 7850 (Model 8422 A) single-quadrupole ICP-MS (Agilent, Singapore). A peristaltic pump connected to an Agilent MicroMist glass concentric nebuliser and an Agilent SPS4 autosampler were used to introduce samples into the instrument. All elements, except B, were measured in He mode. Data acquisition was performed using Agilent ICP-MS MassHunter 5.1 (G7201D Build 653.14) software, and the acquired data were processed using Agilent Online ICP-MS software (G7201D Build 653.14) to create a matrix of elemental concentrations.

Calibration was performed using two separate multi-point external calibration curves, tailored to the elemental abundance: (1) For major elements (Na, Mg, Ca, Fe, K), calibration was prepared using the Agilent environmental calibration standard (p/n 5183-4688), up to 200 mg/L, and (2) for the rest of the elements, calibration was prepared by combining multiple Agilent standards (p/n 8500-6944, 8500-6940, 8500-6942), up to 400 µg/L. A calibration blank was also included in the worklist. All calibration standards were freshly prepared in 2% nitric acid and 0.5% hydrochloric acid in deionised water. A complete list of the monitored elements, along with their natural abundances, is provided in Table S6.

The instrument was tuned daily before analysis using a tune solution prepared by diluting the ICP-MS stock tune solution (10 µg/mL) to 1.00 µg/L in 2% nitric acid. Additionally, a 100 µg/L solution of indium and bismuth was automatically infused during data acquisition as an internal standard. Analytical signals were normalized to the internal standard to correct for instrumental drift and matrix effects.

To ensure method accuracy and reliability, a CRM consisting of organic soybean meal was analysed in duplicate at the start and end of each batch, and recovery rates for elements with manufacturer-reported concentrations (Cu, Al, K, Fe, Ca, Mg, Na, B, Zn, and Mn) were monitored. Spike recovery experiments were performed on a soybean sample to evaluate potential matrix effects. Spiked samples were prepared in triplicate, and recoveries were calculated by comparing the measured concentrations with the expected values. Elemental stability over time was assessed by comparing CRM element concentrations at the start and end of each sample batch across multiple batches and days. Precision was evaluated by assessing intra-day repeatability and inter-day reproducibility using two standard mixes: one containing Na, Mg, Ca, Fe, and K at 195 µg/L and another containing the remaining elements at 3.12 µg/L. Three replicate measurements were performed consecutively on the same day and on three separate days. Precision was expressed as the RSD. To minimise bias and systematic errors, samples from different origins were distributed across batches and blanks were included in each batch. IDLs were calculated as three times the standard deviation of ten replicate calibration blanks, while MDLs were derived by applying the appropriate dilution factor from the sample preparation protocol.

### Multivariate analysis

MVA was performed using SIMCA 18 (Umetrics, Umea, Sweden). Prior to the analysis, log 10 normalization was applied to element concentrations to standardize the magnitude of variables and make them more comparable. Before normalization, raw data were screened for missing values and outliers. To explore the structure of the data, PCA was performed as an unsupervised technique, providing insights into patterns, clustering, and potential outliers without relying on classes^49^. To further differentiate between predefined groups, such as samples from different countries, OPLS-DA models were generated. OPLS-DA is a supervised method that removes variation from the descriptor variables unrelated to class distinction, focusing on maximizing the differences between groups and improving classification performance^50^.

For model validation, the dataset was split into training and test sets using a stratified random sampling approach. Specifically, within each country group, a random number was assigned to each sample, and samples were then sorted by these random numbers. The first 20% of samples (lowest random values) from each group were assigned to the test set, and the remaining 80% to the training set. This procedure ensured proportional representation of each country class in both sets, maintaining class balance and reducing bias. This approach is well-established in chemometric and classification studies, providing a robust framework for model development and unbiased independent validation. Confusion matrices derived from the test set were evaluated to assess classification performance, ensuring that the model’s performance was not limited to the training data alone.

Several key metrics were used to evaluate the quality and performance of the model. R^2^X represents the fraction of the variance in the descriptor variables (element concentrations) explained by the model, indicating how well the input data are captured by the model. R^2^Y indicates the proportion of variance in the response variables (country classification) that is explained by the model, reflecting how well the model accounts for the differences between countries. Q^2^ represents the predictive power of the model, indicating how likely the model is to perform well on new, unseen data^51^. A high R^2^X and R^2^Y combined with a high Q^2^ suggests a robust model with strong explanatory and predictive power.

To further assess the robustness of the model and avoid overfitting, permutation tests (*n* = 100) were conducted. This procedure involves randomizing the class labels and rebuilding the model to see if the original model performs significantly better than models with random class assignments. A successful permutation test indicates that the OPLS-DA model’s separation between groups is not due to chance. Cross-validation (7-fold) was also employed during model training to assess internal consistency.

## Supplementary information

Supplementary material

## Data Availability

The authors declare that all relevant data supporting this study has been included in the paper and supplementary materials. The raw dataset generated are not publicly available but are available from the corresponding author on reasonable request.
