# Supplementary material for: Mapping the global origins of soybean: a study using ICP-MS and chemometrics
Source: NPJ Sci Food. 2025 Dec 9;9:263. doi: 10.1038/s41538-025-00630-5 (PMC12689669; doi:10.1038/s41538-025-00630-5)
Supplement: Supplementary file 1 — Supplementary material [file 41538_2025_630_MOESM1_ESM.pdf]

SUPPLEMENTARY MATERIAL

Mapping the Global Origins of Soybean: A Study Using ICP-MS and Chemometrics

M. Mar Aparicio-Muriana[1,a,*], Yunhe Hong[1,a], Cynthia A. Chilaka[1], Brian Quinn[1], Alfredo M. Montes-Niño[2], Nicholas Birse[1], Christopher T. Elliott[1,3]

[1] National Measurement Laboratory: Centre of Excellence in Agriculture and Food Integrity, Institute for Global Food Security, School of Biological Sciences, Queen's University Belfast, United Kingdom

[2] Microbioticos Paraguay SRL, Arsenales & De las Residentas, 111434 San Lorenzo, Paraguay

[3] School of Food Science and Technology, Faculty of Science and Technology, Thammasat University, 99 Mhu 18, Pahonyothin Road, Khong Luang, Pathum Thani 12120, Thailand

[a] These authors contributed equally to this work

*Corresponding author: m.apariciomuriana@qub.ac.uk

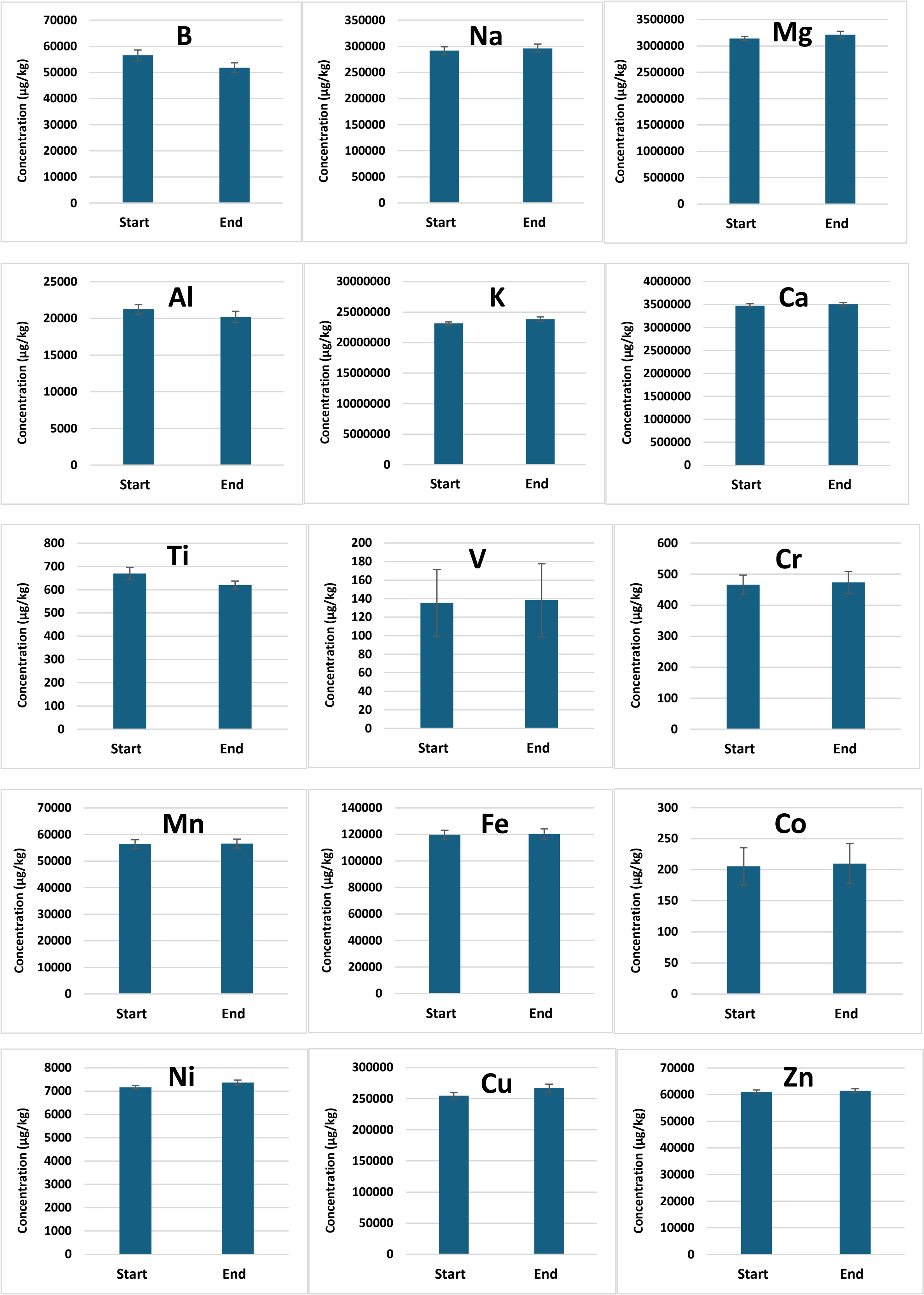

Figure S1. Average concentration of the elements measured in the Certified Reference Material (CRM) at the start and at the end of the sample batches. Bar graphs display the concentration of 40 elements included in the model. Error bars represent the standard error of the measurements, calculated as the standard deviation divided by the square root of the number of measurements that comprise the mean. (Continued on next page)

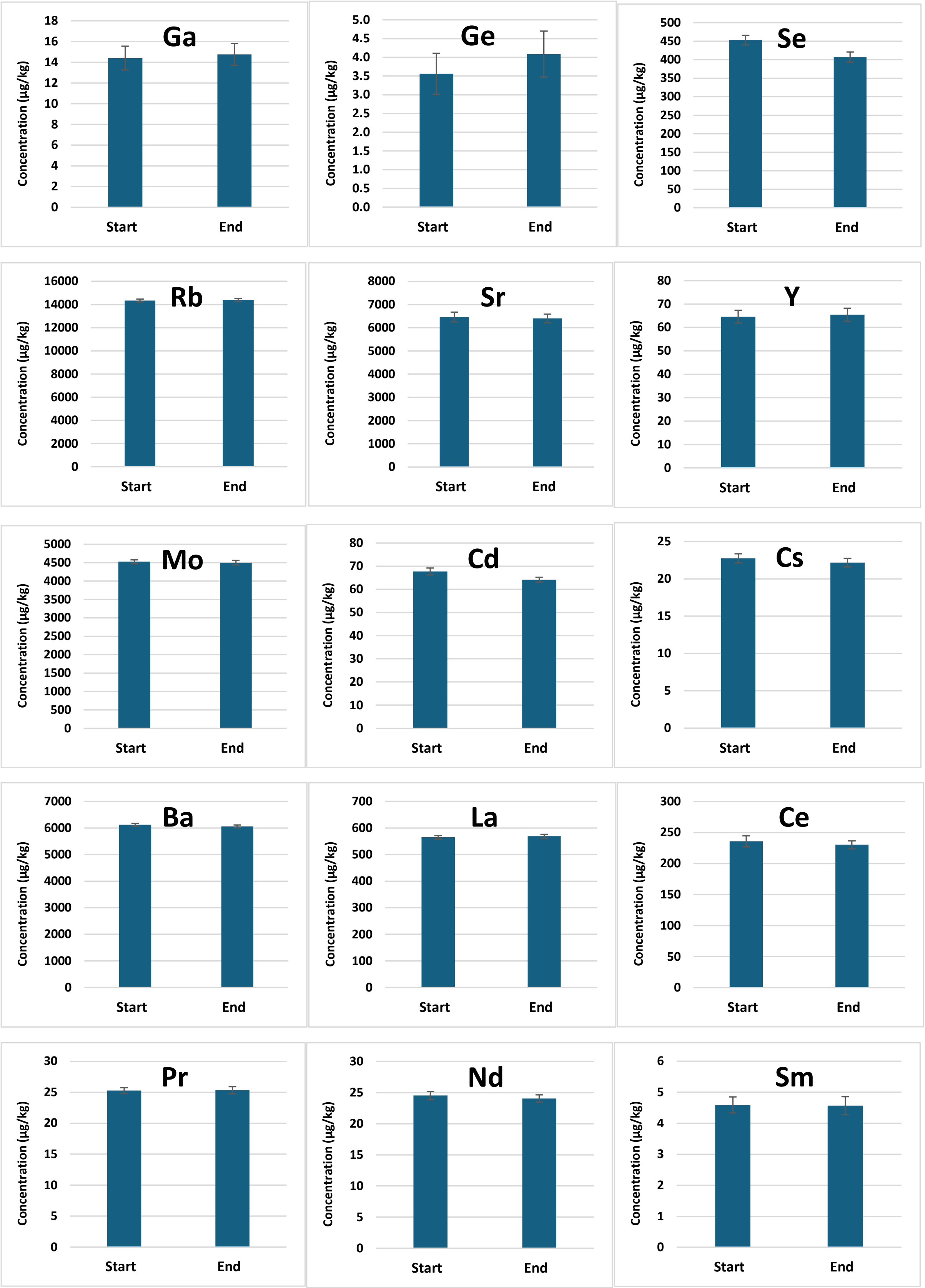

Figure S1. Average concentration of the elements measured in the Certified Reference Material (CRM) at the start and at the end of the sample batches. Bar graphs display the concentration of 40 elements included in the model. Error bars represent the standard error of the measurements, calculated as the standard deviation divided by the square root of the number of measurements that comprise the mean. (Continued on next page)

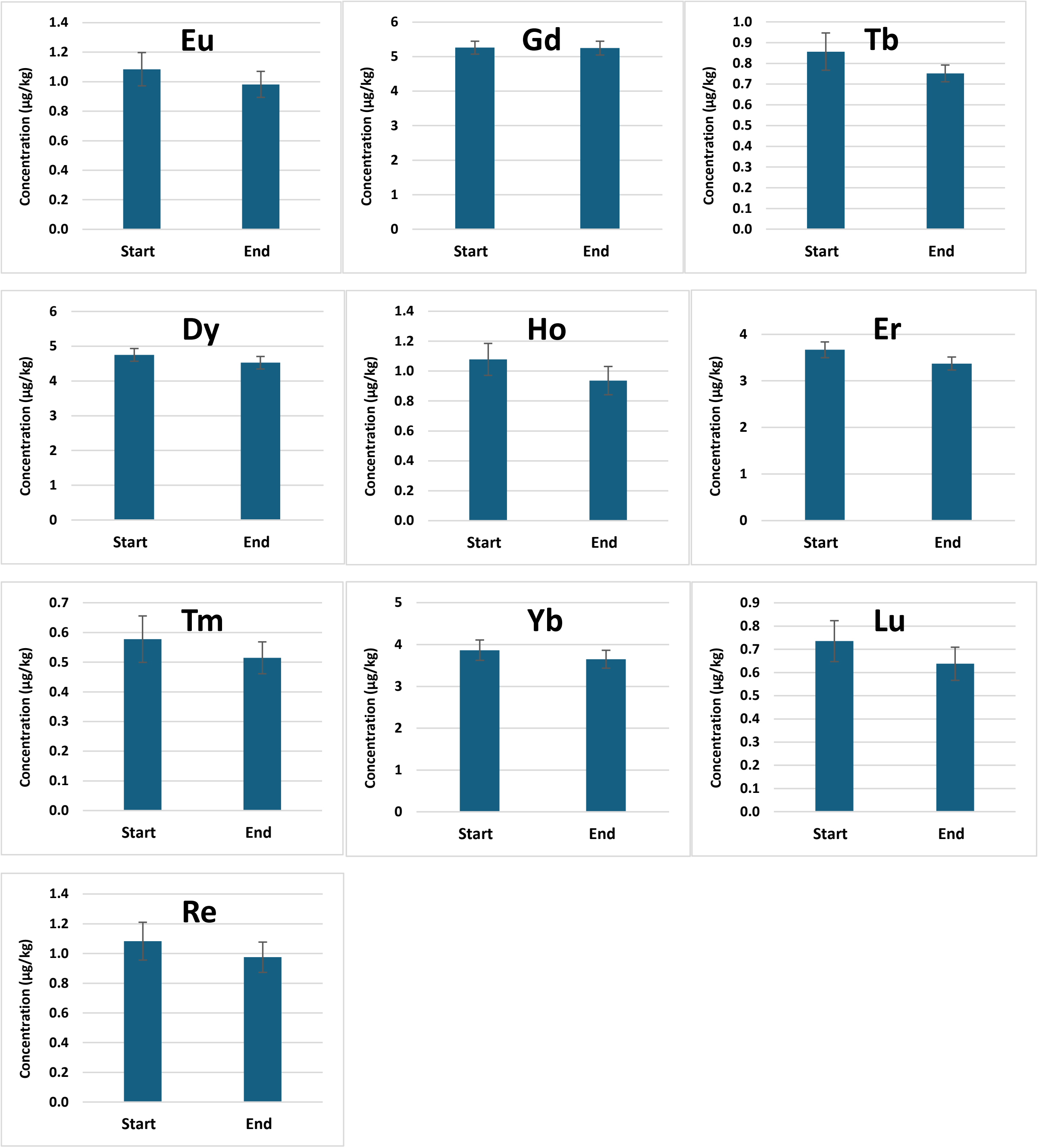

Figure S1. Average concentration of the elements measured in the Certified Reference Material (CRM) at the start and at the end of the sample batches. Bar graphs display the concentration of 40 elements included in the model. Error bars represent the standard error of the measurements, calculated as the standard deviation divided by the square root of the number of measurements that comprise the mean.

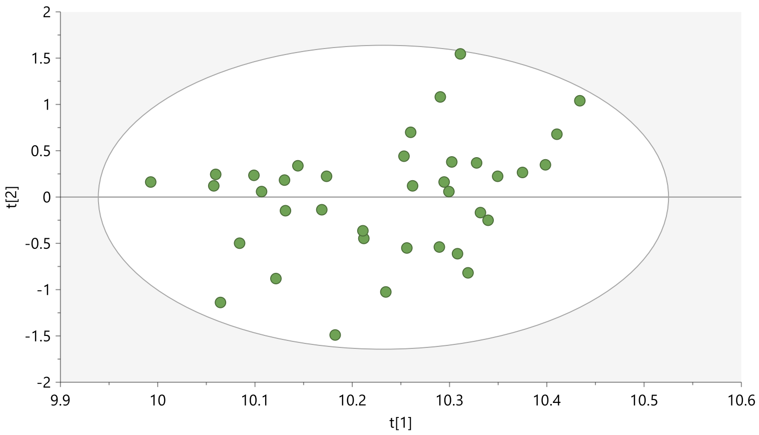

Figure S2. CRM PCA-class score scatter plot (R^2X= 0.996, Q^2=0.995). It illustrates the clustering and consistency of the data, confirming the stability and reliability of the analyses.

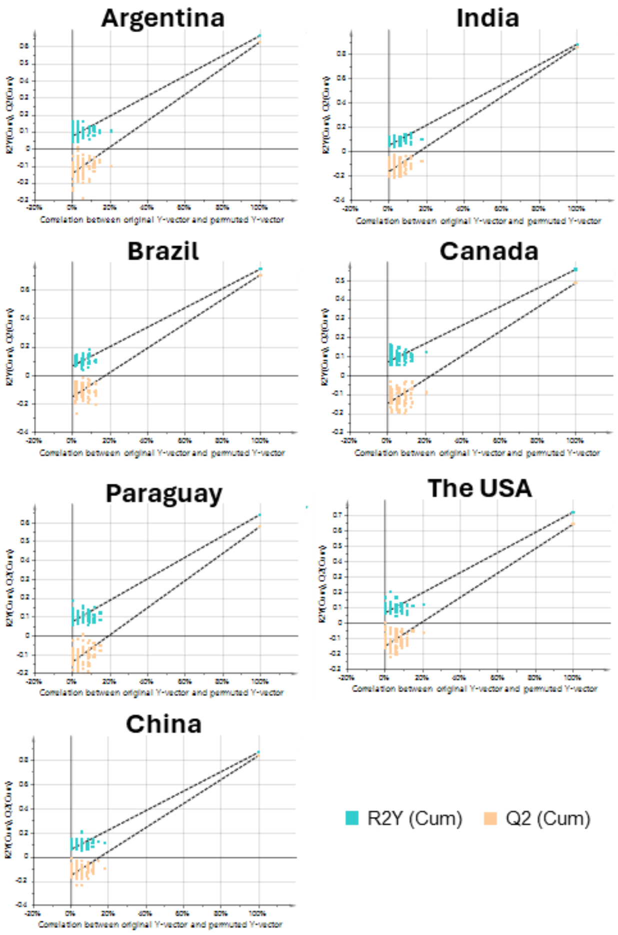

Figure S3. Permutation plot of the OPLS-DA model for soybean classification across seven countries.

Table S1. Spike recovery results of a Canadian spiked soybean sample.

Element	Concentration of unespiked sample (µg/kg)	Spike concentration (µg/kg)	Concentration of spiked sample (µg/kg)	Average recovery, % (n=3)
11 B	2.53 e+04	1.00 e+04	3.09 e+04	87.6
23 Na	4.01 e+03	1.00 e+04	1.21 e+04	86.4
24 Mg	2.34 e+06	1.00 e+06	3.15 e+06	94.3
27 Al	1.18 e+04	1.00 e+03	1.52 e+04	118
39 K	1.74 e+07	1.00 e+06	1.77 e+07	96.2
43 Ca	2.07 e+06	1.00 e+06	2.84 e+06	92.5
47 Ti	624	1.00 e+03	1.66 e+03	102
51 V	28.0	1.00 e+03	1.20 e+03	116
52 Cr	293	1.00 e+03	1.50 e+03	116
55 Mn	2.46 e+04	1.00 e+04	3.59 e+04	104
56 Fe	8.10 e+04	1.00 e+04	9.46 e+04	104
59 Co	65.4	100	186	113
60 Ni	1.20 e+03	100	1.29 e+03	99.3
63 Cu	1.25 e+04	1.00 e+03	1.36 e+04	101
66 Zn	3.87 e+04	1.00 e+03	3.90 e+04	98.4
71 Ga	3.45	10.0	13.2	98.3
72 Ge	0.400	10.0	10.8	104
78 Se	86.5	10.0	92.7	96.1
85 Rb	7.74 e+03	1.00 e+03	8.75 e+03	100
88 Sr	2.68 e+03	1.00 e+03	3.62 e+03	98.3
89 Y	3.92	10.0	14.5	104
95 Mo	1.85 e+03	100	1.97 e+03	101
111 Cd	40.5	10.0	48.5	96.2
133 Cs	8.87	10.0	19.0	101
135 Ba	1.10 e+03	100	1.22 e+03	101
139 La	7.93	1.00	8.73	97.7
140 Ce	17.1	1.00	19.2	106
141 Pr	1.75	1.00	2.92	106
146 Nd	7.69	1.00	8.63	99.3
147 Sm	1.46	1.00	2.12	86.3
153 Eu	0.360	1.00	1.35	99.8
157 Gd	1.21	1.00	2.44	111
159 Tb	0.190	1.00	1.27	107
163 Dy	0.990	1.00	2.04	102
165 Ho	0.230	1.00	1.34	109
166 Er	0.510	1.00	1.67	110
169 Tm	0.0600	1.00	1.13	107
172 Yb	0.340	1.00	1.41	105
175 Lu	0.140	1.00	1.28	112
185 Re	<IDL	1.00	0.950	95.5

Table S2. Percentage differences of analyte concentrations at the start and end of sample batches. Determined as the absolute value of the ratio of the difference between concentrations at the start and end of the batches, divided by their average concentration, and multiplied by 100.

Element	B	Na	Mg	Al	K	Ca	Ti	V	Cr	Mn	Fe	Co	Ni	Cu	Zn	Ga	Ge	Se	Rb	Sr
% Difference	8.81	1.42	2.30	4.95	2.94	0.906	7.74	0.755	1.51	0.280	0.458	2.15	2.81	4.57	0.634	2.50	13.7	10.6	0.391	0.928
**Element**	**Y**	**Mo**	**Cd**	**Cs**	**Ba**	**La**	**Ce**	**Pr**	**Nd**	**Sm**	**Eu**	**Gd**	**Tb**	**Dy**	**Ho**	**Er**	**Tm**	**Yb**	**Lu**	**Re**
% Difference	1.32	0.643	5.40	2.52	0.985	0.645	2.42	0.208	1.97	0.519	9.95	0.240	13.1	4.84	14.1	8.39	11.5	5.69	14.2	10.5

Table S3. Instrument Detection Limits (IDLs) and Method Detection Limits (MDLs). IDLs calculated as three times the standard deviation (SD) of 10 measurements of a calibration blank. MDLs calculated by applying a 200-fold dilution factor to the IDLs.

Element	Mode	ISTD	IDL (µg/kg)	MDL (µg/kg)
B	No gas	209 Bi	0.325	65.0
Na	He	115 In	0.798	160
Mg	He	209 Bi	0.278	55.5
Al	He	115 In	0.314	62.9
K	He	115 In	2.48	496
Ca	He	115 In	11.2	2240
Ti	He	209 Bi	0.261	52.2
V	He	209 Bi	9.10 e-03	1.82
Cr	He	209 Bi	6.24 e-03	1.25
Mn	He	209 Bi	2.42 e-02	4.85
Fe	He	209 Bi	0.147	29.4
Co	He	209 Bi	2.98 e-03	0.597
Ni	He	209 Bi	2.92 e-02	5.84
Cu	He	209 Bi	2.41 e-02	4.83
Zn	He	209 Bi	0.131	26.2
Ga	He	209 Bi	6.03 e-03	1.21
Ge	He	209 Bi	1.50 e-02	3.00
Se	He	209 Bi	8.75 e-02	17.5
Rb	He	115 In	9.72 e-03	1.94
Sr	He	115 In	5.51 e-03	1.10
Y	He	115 In	1.70 e-03	0.341
Mo	He	209 Bi	3.69 e-03	0.738
Cd	He	209 Bi	2.47 e-03	0.494
Cs	He	115 In	1.55 e-03	0.310
Ba	He	115 In	1.37 e-02	2.73
La	He	115 In	1.55 e-03	0.310
Ce	He	115 In	1.45 e-03	0.290
Pr	He	115 In	9.49 e-04	0.190
Nd	He	115 In	1.55 e-03	0.310
Sm	He	115 In	1.26 e-03	0.253
Eu	He	115 In	1.58 e-03	0.316
Gd	He	115 In	1.58 e-03	0.316

Tb	He	115 In	1.45 e-03	0.290
Dy	He	115 In	1.55 e-03	0.310
Ho	He	115 In	1.45 e-03	0.290
Er	He	115 In	1.45 e-03	0.290
Tm	He	115 In	1.45 e-03	0.290
Yb	He	115 In	9.49 e-04	0.190
Lu	He	115 In	1.26 e-03	0.253
Re	He	209 Bi	1.26 e-03	0.253

Table S4. Overview of state origins, sample sizes, and grouping strategy for soybeans from the USA.

Sample number	State of origin	Regional groups
9	Illinois	Central
10	Nebraska	Central
5	Iowa	Central
22	South Dakota	Central
1	North Dakota	Central
5	Kansas	Central
6	Missouri	Central
2	Arkansas	Central
2	Ohio	East
2	Maryland	East
3	North Carolina	East

Table S5. Overview of state origins, sample sizes, and grouping strategy for soybeans from Brazil.

Sample number	State of origin	Regional groups
10	Minas Gerais	Cerrado
1	Federal District	Cerrado
5	Goiás	Cerrado
7	Saõ Paulo	Atlantic Forest
7	Paraná	Atlantic Forest
1	Rio Grande do Sul	Atlantic Forest

Table S6. Monitored elements and their natural abundance.

^{mass}Element	^{11}B	^{23}Na	^{24}Mg	^{27}Al	^{39}K	^{43}Ca	^{47}Ti	^{51}V
Abundance (%)	80.1	100	79.0	100	93.3	0.135	7.44	99.8
^{mass}Element	^{52}Cr	^{55}Mn	^{56}Fe	^{59}Co	^{60}Ni	^{63}Cu	^{66}Zn	^{71}Ga
Abundance (%)	83.8	100	91.8	100	26.2	69.2	27.9	39.9
^{mass}Element	^{72}Ge	^{78}Se	^{85}Rb	^{88}Sr	^{89}Y	^{95}Mo	^{111}Cd	^{133}Cs
Abundance (%)	27.5	23.8	72.2	82.6	100	15.9	12.8	100
^{mass}Element	^{135}Ba	^{139}La	^{140}Ce	^{141}Pr	^{146}Nd	^{147}Sm	^{153}Eu	^{157}Gd
Abundance (%)	6.52	99.9	88.5	100	17.2	15.0	52.2	15.7
^{mass}Element	^{159}Tb	^{163}Dy	^{165}Ho	^{166}Er	^{169}Tm	^{172}Yb	^{175}Lu	^{185}Re
Abundance (%)	100	24.9	100	33.6	100	21.8	97.4	37.4
